# Long-Term Dietary Patterns Are Reflected in the Plasma Inflammatory Proteome of Patients with Inflammatory Bowel Disease

**DOI:** 10.3390/nu14122522

**Published:** 2022-06-17

**Authors:** Arno R. Bourgonje, Laura A. Bolte, Lianne L. C. Vranckx, Lieke M. Spekhorst, Ranko Gacesa, Shixian Hu, Hendrik M. van Dullemen, Marijn C. Visschedijk, Eleonora A. M. Festen, Janneke N. Samsom, Gerard Dijkstra, Rinse K. Weersma, Marjo J. E. Campmans-Kuijpers

**Affiliations:** 1 Department of Gastroenterology and Hepatology, University Medical Centre Groningen, University of Groningen, Hanzeplein 1, 9713 GZ Groningen, The Netherlands; a.r.bourgonje@umcg.nl (A.R.B.); l.a.bolte@umcg.nl (L.A.B.); l.l.c.vranckx@umcg.nl (L.L.C.V.); l.m.spekhorst@umcg.nl (L.M.S.); r.gacesa@umcg.nl (R.G.); dhu.sxhu@hotmail.com (S.H.); h.m.dullemen@umcg.nl (H.M.v.D.); m.c.visschedijk@umcg.nl (M.C.V.); e.a.m.festen@umcg.nl (E.A.M.F.); gerard.dijkstra@umcg.nl (G.D.); r.k.weersma@umcg.nl (R.K.W.); 2 Department of Pediatrics, Division of Gastroenterology, Erasmus University Medical Centre, 3000 CA Rotterdam, The Netherlands; j.samsom@erasmusmc.nl

**Keywords:** diet, Crohn’s disease, ulcerative colitis, proteomics, FGF-19

## Abstract

Diet plays an important role in the development and progression of inflammatory bowel disease (IBD, comprising Crohn’s disease (CD) and ulcerative colitis (UC)). However, little is known about the extent to which different diets reflect inflammation in IBD beyond measures such as faecal calprotectin or C-reactive protein. In this study, we aimed to unravel associations between dietary patterns and circulating inflammatory proteins in patients with IBD. Plasma concentrations of 73 different inflammation-related proteins were measured in 454 patients with IBD by proximity extension assay (PEA) technology. Food frequency questionnaires (FFQ) were used to assess habitual diet. Principal component analysis (PCA) was performed to extract data-driven dietary patterns. To identify associations between dietary patterns and plasma proteins, we used general linear models adjusting for age, sex, BMI, plasma storage time, smoking, surgical history and medication use. Stratified analyses were performed for IBD type, disease activity and protein intake. A high-sugar diet was strongly inversely associated with fibroblast growth factor-19 (FGF-19) independent of IBD type, disease activity, surgical history and deviance from recommended protein intake (false discovery rate (FDR) < 0.05). Conversely, a Mediterranean-style pattern was associated with higher FGF-19 levels (FDR < 0.05). A pattern characterised by high alcohol and coffee intake was positively associated with CCL11 (eotaxin-1) levels and with lower levels of IL-12B (FDR < 0.05). All results were replicated in CD, whereas only the association with FGF-19 was significant in UC. Our study suggests that dietary habits influence distinct circulating inflammatory proteins implicated in IBD and supports the pro- and anti-inflammatory role of diet. Longitudinal measurements of inflammatory markers, also postprandial, are needed to further elucidate the diet–inflammation relationship.

## 1. Introduction

Inflammatory bowel diseases (IBD), comprising Crohn’s disease (CD) and ulcerative colitis (UC), are chronic immune-mediated diseases of the gastrointestinal (GI) tract, involving a complex interplay between host genetics, dysregulated immune responses, the gut microbiome and environmental factors, including diet. IBD is characterised by considerable heterogeneity, which complicates the prediction of disease course and effectiveness of therapeutic interventions [1].

Currently available inflammatory biomarkers, including C-reactive protein (CRP) and faecal calprotectin (FCal), are often non-specific and lack discriminative power, illustrating the need for additional surrogate disease biomarkers, which could preferably be applied to patients suffering from subclinical disease activity [2,3]. The emergence of high-throughput proteomics technologies facilitated the pursuit of clinically useful biomarkers in IBD, e.g., for diagnostic purposes, disease activity monitoring, discovery of therapeutic targets and prediction of therapeutic responses [4]. In this context, however, it is critical to carefully examine the relevant determinants of circulating protein biomarkers to enable an adequate approximation of interindividual protein variability.

Lifestyle and cardiovascular factors (e.g., body-mass index (BMI) and blood pressure) are known to have a strong impact on the blood proteome [5]. In this regard, dietary intake may also affect the plasma inflammatory proteome, which has, however, been insufficiently explored in the context of IBD.

Previous studies observed that dietary patterns are strongly associated with a greater likelihood of IBD development [6], pro-inflammatory gut microbial signatures [7], intestinal inflammation and disease exacerbations in established IBD [8,9,10,11]. A myriad of studies linked specific dietary components to inflammatory markers such as CRP or FCal or used various in vitro and in vivo approaches to uncover pro-inflammatory or anti-inflammatory effects [12,13]. While the pro-inflammatory and anti-inflammatory role of specific nutrients is well described, studies on the overall dietary composition are important to generate food-based dietary recommendations [6,14].

In this study, we aim to uncover associations between dietary patterns and the plasma inflammatory proteome in patients with IBD. To do so, high-throughput proteomics technology (Olink Proteomics^®^) was leveraged to quantify 92 inflammation-related plasma proteins in 454 patients with IBD, and these proteins were associated with dietary patterns using data derived from food frequency questionnaires (FFQ).

## 2. Materials and Methods

### 2.1. Study Population

Patients with an established diagnosis of IBD for at least 1 year were included at the outpatient clinic of the University Medical Centre Groningen (UMCG), Groningen, the Netherlands. These patients participated in the 1000IBD project, an extensively phenotyped prospective cohort comprising over 1000 patients with IBD who live in the northern regions of the Netherlands [15]. This study was approved by the Institutional Review Board (IRB) of the University Medical Centre Groningen (UMCG, registered as no. 2008/338). All patients provided written informed consent for the use of their data and biomaterials. The study has been performed in accordance with the principles of the Declaration of Helsinki (2013). Patients who were included in the present study were enrolled between 2010 to 2019 and were selected if they had provided both plasma samples for proteomic analysis and a complete FFQ.

### 2.2. Data Collection and Definitions

Demographic and clinical data of the study population were collected at time of plasma sampling and included age, sex, body-mass index (BMI), smoking status, Montreal disease classification, medication use, surgical history and disease activity. Clinical disease activity was recorded using the Harvey–Bradshaw Index (HBI) for patients with CD and the Simple Clinical Colitis Activity Index (SCCAI) for patients with UC. Blood C-reactive protein (CRP) concentrations were measured routinely by nephelometry at the time of plasma withdrawal. CRP was recorded as a binary variable since many values did not pass the lower limit of detection and were reported using routinely applied thresholds (<5 mg/L or 0.3 mg/L from 2016 onwards). In stratified analyses for disease activity (vide infra), quiescent disease was defined as having both low clinical disease activity scores (HBI score < 5 in case of CD and SCCAI score ≤ 2 in case of UC) and CRP levels < 5 mg/L, while remaining patients were classified as having ‘active disease’.

### 2.3. Proteomic Profiling

Using proximity extension assay (PEA) technology, plasma concentrations of 92 different (mainly inflammation-related) proteins were quantified (ProSeek Multiplex Inflammation panel, *Olink Proteomics*^®^, Uppsala, Sweden). Full names, detection rates and corresponding UniProt IDs of these proteins can be found in Table S1. Proteomic profiling was performed in the *Olink*^®^ testing facility in Uppsala, Sweden, through the incubation of 92 matched oligonucleotide-labelled antibody probes with the samples, which enabled pair-wise binding to the target proteins within each sample. When two probes of the same type co-localized in close proximity, hybridization occurred, which was followed by DNA polymerase extension. Ultimately, the specific DNA sequence was detected and amplified using real-time microfluidic quantitative polymerase chain reaction (rt-qPCR, Biomark HD Instrument, Fluidigm^®^, San Francisco, CA, USA) [16]. With the goal of reducing technical variation, plasma samples were randomized on the experimental plates using an algorithm that included randomization by patient age, sex and IBD type (either CD or UC). Subsequently, an inter-plate intensity normalisation procedure was performed prior to data analysis, which is a method that uses the overall median of the experiment as primary normalisation factor. Finally, measured protein concentrations were normalised on a log2-scale, where values were obtained from the inverted Ct-values of the rt-qPCR procedure and were expressed as normalised protein expression (NPX) values. The NPX unit represents relative quantification and thus constitutes an arbitrary unit, which precluded the analysis of absolute values between different proteins, while allowing comparative analyses for each individual protein across different samples. Samples that delivered values deviating >0.3 NPX from the median of internal controls were excluded from the analyses and flagged as QC-failed (*n* = 40). In addition, 18 of the total 92 proteins demonstrated a low rate of detection (<30%) and were also excluded from further analysis across all samples (Table S1). Furthermore, values for the tumour necrosis factor alpha (TNF-α) protein were removed from the analyses as the measurements of this protein were technically perturbed by anti-TNF-α antibodies-bound TNF-α (e.g., infliximab [IFX] or adalimumab [ADA]). As the *Olink*^®^ assay used within this study (reference no. 95302) contained polyclonal antibodies against TNF-α that also detect monomeric forms, the concurrent detection of biologically inactive forms of TNF likely resulted in suboptimal measurements [17,18]. Finally, NPX values that fell below a protein-specific detection limit were designated as missing values, since their inclusion in the analysis did not alter the results. After these data processing steps, 73 different plasma protein levels were available for analysis for a total of 467 patients with IBD.

### 2.4. Dietary Data

Habitual dietary intake was evaluated using semi-quantitative FFQs, which have been developed and validated by the Department of Human Nutrition of Wageningen University, the Netherlands [19]. Patients were requested to complete the FFQ using their daily dietary intake over the past month as a reference period. Naturally occurring portions and commonly employed household measures were used to estimate portion sizes. Intake in grams per day was calculated by converting frequencies of consumption into daily equivalents and multiplying by portion sizes. The Dutch food composition table (NEVO 2011, RIVM, Bilthoven, The Netherlands) was used to determine the daily nutrient intake. The obtained 110 individual food items were collapsed into 26 standardised food groups for principal component analysis (Table S2).

**Figure 1:**
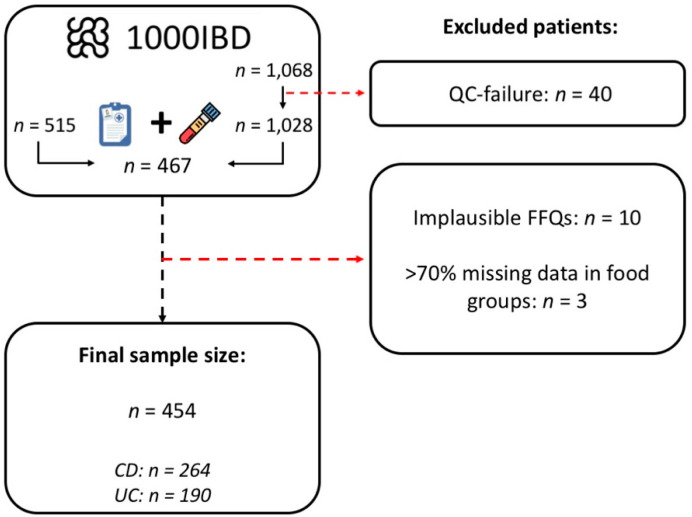
**Flowchart of the study inclusion and exclusion of participants.** In total, proteomics data were generated for 1028 patients who participated in the 1000IBD project, while for 515 patients FFQs were available. After cross-referencing both datasets, overlapping data were available for 467 patients. After excluding FFQs with implausible energy intake or missing food items, a final total of 454 patients were eligible for analysis in the present study. Abbreviations: CD, Crohn’s disease; FFQ, food frequency questionnaire; QC, quality control; UC, ulcerative colitis.

To account for over- and underreporting inherent to FFQs, we excluded 10 participants with implausible energy intake, defined as values outside of the range of 500–3500 kcal/day for women and 800–4000 kcal/day for men [20]. Caloric intakes over 4000 were considered plausible for 4 participants given their anthropometric data and basal metabolic rate, BMR (Table S3). BMR was estimated using age- and sex specific Harris–Benedict equations. A ratio of energy intake to BMR (EI/BMR) in the range of 1.35–2.4 was considered a normal-reporting of dietary intake [21]. We excluded 3 participants with >70% missing data in food groups required for the PCA, leaving a total of 454 FFQs for analysis (Figure 1).

### 2.5. Statistical Analysis

#### 2.5.1. General Descriptive and Inferential Statistics

Demographic and clinical characteristics of the study population were presented as means ± standard deviation (SD), medians with interquartile range [IQR] or as proportions *n* with corresponding percentages (%). Normality assessment was performed by visual inspection of normal probability (Q–Q) plots and histograms. Differences between groups were tested using independent sample *t*-tests or Mann–Whitney *U*-tests in the case of non-normally distributed continuous variables, while categorical variables were compared between groups using Pearson’s chi-square tests or Fisher’s exact tests, as appropriate. Two-tailed *p*-values < 0.05 were considered statistically significant. Statistical analyses were performed using R (v.3.5.2, Vienna, Austria). Data visualisation was performed with Python (v.3.8.5, Python Software Foundation, Wilmington, DE, USA) using the *pandas* (v.1.3.3), *matplotlib* (v.3.4.3) and *seaborn* (v.0.11.2) packages.

#### 2.5.2. Principal Component Analysis

Principal component analysis (PCA) was performed with orthogonal (*varimax*) rotation on the 26 food groups to derive a posteriori identified dietary patterns, creating combinations of food groups in order to explain the maximum degree of variance in a given correlation matrix. Component scores were generated per individual patient, which represent the sum of the products of the correlation strength of each food group with the overall intake registered for that patient. As such, individual patients can be ranked by their component scores based on their consumption of foods from groups that are highly weighted in that specific component. Orthogonal rotation was performed to achieve optimal interpretability of the extracted components (dietary patterns). Factorizability of the food group data was checked using a correlation matrix, Barlett’s test of sphericity and the Kaiser–Meyer–Olkin test. Scree plots and interpretability criteria were used to determine the number of extracted components (dietary patterns) to retain (Figures S1 and S2). Coefficient scores with absolute values < −0.3 or >0.3 were considered relevant [8]. Finally, for each patient, component scores were calculated per dietary pattern by the sum of the food groups weighted by their loading factors.

#### 2.5.3. Permutational Analysis of Variance

First, the total protein variance explained by the top five principal components, i.e., dietary patterns, was calculated. For this analysis, missing values in protein levels were imputed using half of the protein-specific lower limit of detection. Next, a permutational univariate analysis of variance was performed as implemented in the *adonis* function of the R package *vegan* (v.2.4-6) using 1000 permutations and Euclidean distances of the relative quantifications of plasma proteins.

#### 2.5.4. Association Analyses

To determine whether a higher adherence to the identified dietary patterns is associated with specific plasma proteins, we constructed four sets of multivariate generalised linear models. In each case, one model was performed per dietary pattern versus each plasma protein, correcting for age, sex, BMI, plasma storage time, smoking status, disease activity and in models stratified for IBD type, adding current medications and surgical history, represented as:

*GLM*: *Protein* ~ *intercept* + *Diet PC* + *Age* + *Sex* + *BMI* + *Plasma storage time* + *Smoking* + *Disease activity* + *Current medications* + *Surgical history*

The first set of models was performed in the full IBD cohort (*n* = 454). Subsequently, the analysis was performed stratified by CD (*n* = 264) and UC (*n* = 190) while adjusting for disease-specific clinical scores, medication use and surgical history (Supplementary Methods). Finally, we stratified according to disease activity, i.e., separating patients with quiescent (*n* = 239) and active disease (*n* = 200) [22] and according to protein consumption deviating from recommended intake [8], i.e., separating patients with sufficient (*n* = 256) and insufficient (*n* = 193) protein intake in grams per kg of bodyweight. Protein intake below the recommended daily allowance (0.8 g/kg in remission and 1.2 g/kg for active disease) was deemed as insufficient intake [14,23]. All analyses were adjusted for multiple testing using the Benjamini–Hochberg method as implemented in the *p.adjust* function in R. Subsequently, significance was considered under a false discovery rate (FDR) of 5% (0.05). This approach allows us to reduce the number of false discoveries derived from multiple hypothesis testing. Detailed descriptions of the models performed are provided in the Supplementary Methods.

## 3. Results

### 3.1. Cohort Description

In total, 454 patients were included, of whom 264 (58.1%) were diagnosed with CD and 190 (41.9%) with UC. Patient demographic and clinical characteristics are presented in Table 1. Patients with CD were younger (39.2 ± 14.1 vs. 44.5 ± 14.2 years, *p* < 0.01) and more often female (67.0% vs. 54.7%, *p* < 0.01), had a lower BMI (23.9 (21.4–27.6) vs. 25.5 (22.8–29.1), *p* < 0.01) and smoked more frequently (28.4% vs. 10.9%, *p* < 0.01) compared to patients with UC. Regarding medication use, patients with CD more often used TNF-α-antagonists, thiopurines and methotrexate (all *p* ≤ 0.01), while patients with UC more frequently used aminosalicylates (*p* < 0.01). The proportion of patients who underwent ileocecal resection was higher in the CD compared to the UC group (*p* < 0.01), whereas the history of (partial) colon resections was comparable between groups (*p* = 0.76). Finally, elevated CRP levels (>5 mg/L, tested binary) were more frequent in CD compared to UC patients (31.7% vs. 20.3%, *p* < 0.05). According to our definition, 239 patients (54.4%) were in remission, and 200 patients (45.6%) had active disease.

### 3.2. Habitual Dietary Intake

Summary statistics of the dietary intake are presented in Table 2. Patients with CD had a lower intake of total protein as well as animal protein in grams per day compared to UC patients (total protein: 63.6 (52.2–76.5) vs. 70.6 (57.5–83.5) g/day, *p* = 0.001; animal protein: 36.1 (28.2–45.8) vs. 41.1 (32.2–49.5) g/day, *p* = 0.003). Protein intake was overall low in our cohort (65.7 (54.3–80.5) g/day). Therefore, we also calculated the protein intake in grams per kg body weight as an indicator of protein insufficiency. Protein intake was insufficient, i.e., below the recommended daily allowance, for 33.9% of patients in remission (<0.8 g/kg body weight) and for 85.9% of participants with active disease (<1.2 g/kg body weight). In terms of food groups, patients with CD consumed fewer dairy products and more non-alcoholic beverages compared to UC patients (all FDR < 0.05). All other food groups and nutrients were comparable between CD and UC.

### 3.3. Data-Driven Identification of Dietary Patterns

To identify data-driven dietary patterns in our cohort, principal component analysis (PCA) was performed on 26 food groups. Factorizability of the food group dataset was tested and observed to be appropriate for PCA (Bartlett’s test of Sphericity: *p* < 0.001; Kaiser–Meyer–Olkin test, measure of sample adequacy (MSA): 0.64). None of the food groups had MSA values < 0.5. A scree plot was created to determine the number of patterns to retain (Figure S1).

The first five principal components (with eigenvalues ≥ 1) demonstrated a proportional variance of 10%, 8%, 8%, 7% and 7%, respectively, cumulatively explaining 40% of the total variation in the dietary data. Loading factors (representing the weights of individual food groups within each dietary pattern) were extracted from the PCA in order to characterise the different dietary patterns, where values < −0.3 and > 0.3 were considered relevant (Table 3, Figure S2). The first dietary pattern (PC1) was characterised by a high intake of bread, spreads, pastry, sugar and sweets, savoury snacks and juice, foods that are high in refined carbohydrates [23]. The second dietary pattern (PC2) was marked by a high intake of healthy foods including fruit, fish, nuts, eggs, tea and cereals and a low intake of meat and non-alcoholic beverages, resembling a traditional Mediterranean-style dietary pattern [24]. The third dietary pattern (PC3) revealed high intake of sauces, pasta, ready-made meals, rice, alcohol, nuts and savoury snacks, i.e., processed or ready-made foods [24]. The fourth dietary pattern (PC4) was characterised by an exceptionally high intake of coffee and alcohol (highest loaded food groups) followed by spreads and dairy as opposed to a low intake of tea [24]. The fifth dietary pattern (PC5) was composed of a high intake of vegetables, potatoes, meat and legumes, resembling a ‘Traditional Dutch’ pattern as previously reported in cohorts from different regions in the Netherlands [8]. Robust PCA confirmed the existence of patterns derived from PCA with *varimax* rotation with, as expected, lower factor loadings (Supplementary Results and Table S4).

### 3.4. Associations between Dietary Intake Patterns and Plasma Protein Levels

To test which dietary pattern had the highest influence on overall protein variation, we started with a permutational analysis of variance. The PC that explained most variation in protein levels was PC4 (*p* = 0.019, R^2^ = 0.5%). In descending order of explained variance, PC4 was followed by PC5, PC1, PC2 and PC3. However, none of the patterns significantly explained the variation in protein levels after multiple testing adjustments (FDR > 0.05, Table S5). Since dietary choices can be affected by symptoms such as diarrhoea, we also compared PC loadings between patients with and without active disease and surgery. The identified dietary patterns did not significantly differ between patients with active disease compared to those with quiescent disease at time of sampling, nor between patients with or without surgical history (Table S6).

**Figure 2:**
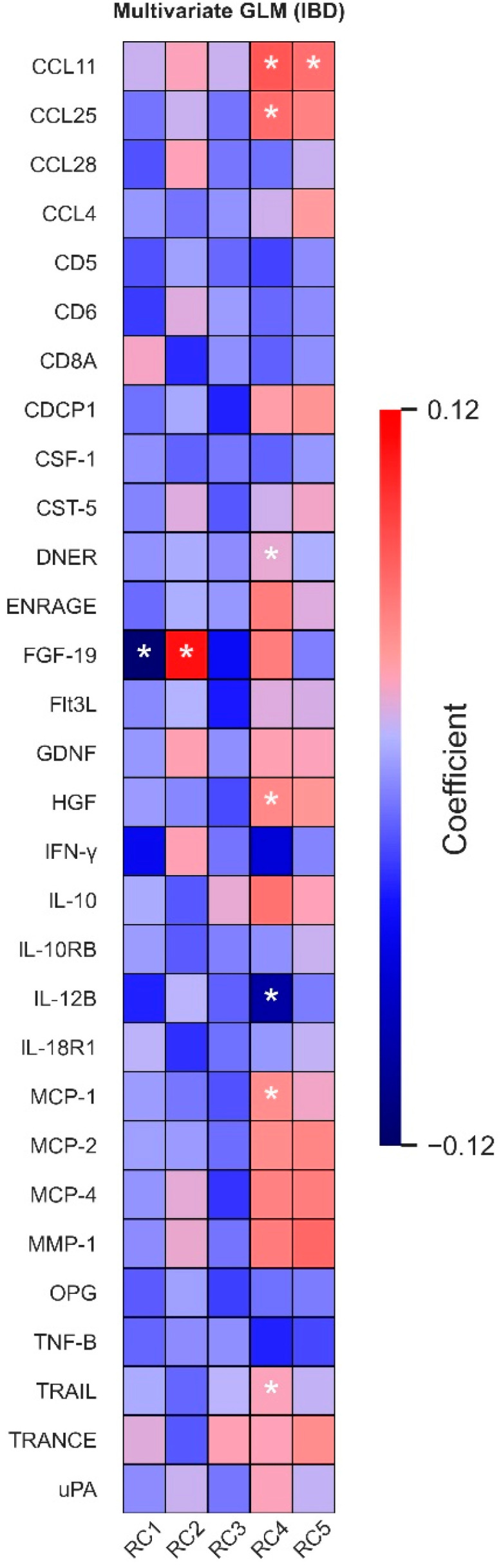
**Associations between dietary intake patterns and plasma protein levels in patients with IBD.** The heatmap indicates the size of the beta coefficients of the studied associations, only showing proteins that had at least one nominally significant association (*p* < 0.05) with a dietary pattern. The first dietary pattern (RC1) was rich in refined carbohydrates, characterised by high intake of bread, spreads, pastry, sugar and sweets, savoury snacks and juice. The second dietary pattern (RC2) was a ‘Mediterranean-style’ dietary pattern, as evidenced by a high intake of fruit, fish, nuts, eggs, tea and cereals and a low intake of meat and non-alcoholic beverages. The third dietary pattern (RC3) was a rather mixed pattern with many processed or ready-made foods, characterised by high intake of sauces, pasta, ready-made meals, rice, alcohol, nuts and savoury snacks. The fourth pattern (RC4) was a typical ‘beverages pattern’, characterised by an exceptionally high intake of coffee and alcohol as well as spreads and dairy, but a low intake of tea. The fifth pattern (RC5) was a ‘Traditional Dutch’ dietary pattern, composed of a high intake of vegetables, potatoes, meat and legumes. Asterisks (*) indicate statistically significant associations (FDR < 0.05). Abbreviations: GLM, general linear model; RC, rotated component.

Next, we associated each individual protein to the five PCA-derived dietary patterns in a multivariate analysis of the full IBD cohort (FDR < 0.05) (Figure 2). PC1, characterised by high intake of refined carbohydrates, was inversely associated with plasma levels of fibroblast growth factor-19 (FGF-19), whereas a positive association was observed between FGF-19 and the Mediterranean-style pattern (PC2). The pattern mostly characterised by a high alcohol and coffee intake (PC4) showed positive associations with several inflammatory proteins, including C-C motif chemokine 1 (CCL11, a.k.a. eotaxin-1), C-C motif chemokine 25 (CCL25), monocyte chemotactic protein 1 (MCP-1), hepatocyte growth factor (HGF), TNF-related apoptosis-inducing ligand (TRAIL) and Delta and Notch-like epidermal growth factor-related receptor (DNER). Conversely, the same pattern showed a negative association with interleukin-12 subunit beta (IL-12B) levels. Finally, the ‘Traditional Dutch’ dietary pattern (PC5) was positively associated with plasma CCL11 levels.

Associations remained significant when analysing CD patients separately (all FDR < 0.05). In addition, the pattern high in alcohol and coffee (PC4) showed positive associations with several monocyte chemotactic proteins (MCP-1, -2 and -4), extracellular newly identified receptor for advanced glycation end products binding protein (EN-RAGE, a.k.a. S100A12 or calgranulin C) and urokinase-type plasminogen activator (uPA) (FDR < 0.05). In patients with UC, the association between PC1 and plasma FGF-19 levels was the only significant association (FDR < 0.05) (Table S7).

### 3.5. Disease Activity Affects Most Dietary Pattern–Protein Associations, except for FGF-19

**Figure 3:**
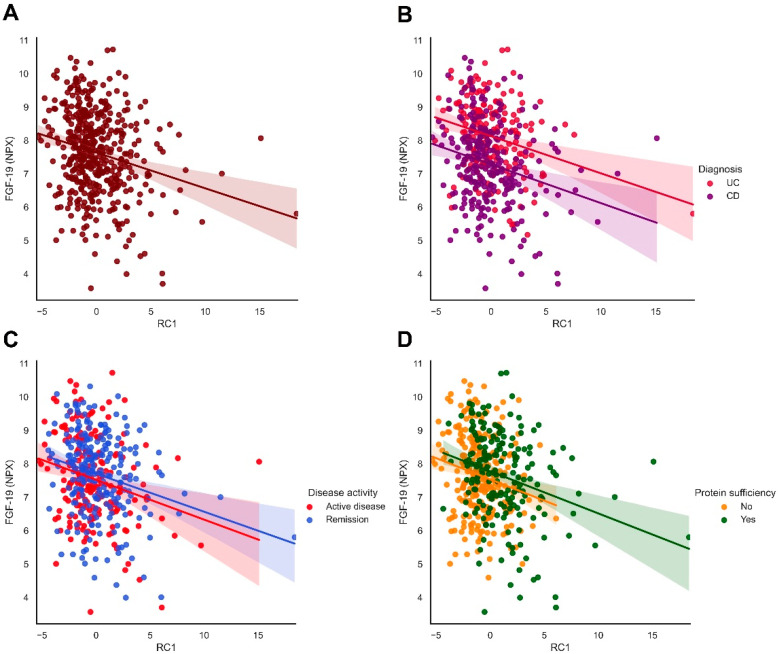
Plasma FGF-19 levels are strongly associated with the first dietary pattern (PC1), mainly representing a diet rich in refined carbohydrates. (**A**) Plasma FGF-19 levels are inversely correlated with RC1. (**B**–**D**) The association between plasma FGF-19 levels and RC1 remained robust throughout all analyses, including when stratified by diagnosis (CD or UC, panel B), disease activity (panel C) or protein intake (panel D). Abbreviations: CD, Crohn’s disease; FGF-19, fibroblast growth factor-19; RC1, rotated component 1; UC, ulcerative colitis.

Since disease activity at the time of sampling may influence the observed associations between dietary patterns and plasma protein levels, we subsequently performed a stratified analysis for disease activity. In patients with quiescent disease, PC1 remained significantly inversely associated with plasma FGF-19 levels, whereas an additional negative association emerged with plasma levels of T-cell surface glycoprotein CD6 isoform (CD6). Furthermore, PC4 showed consistent positive associations with CCL11 and CCL25 levels and an inverse association with IL-12B levels. In patients with active disease, PC1 still remained significantly associated with FGF-19 levels, as well as PC2 and FGF-19 (both FDR < 0.05). The association between PC1 and plasma FGF-19 levels was most robust, i.e., remained significant in all stratified analyses (Figure 3).

### 3.6. Protein Intake Influences Associations between Dietary Patterns and Plasma Proteins

Finally, since protein insufficiency has been reported to affect the clinical course of IBD [8] and protein intake was relatively low in our cohort (Table 2), we stratified according to sufficient and insufficient protein intake. Here, we still observed the previously identified associations between plasma FGF-19 levels and PC1 and PC2, while the inverse association between FGF-19 and PC1 was less significant in patients with insufficient protein intake (FDR = 0.067), who showed more pronounced positive associations with CCL11, CDCP1, HGF and inverse associations with IL12B levels. Plasma levels of CDCP1 and HGF were positively associated with PC5, while CCL11 levels showed a positive association with PC4 (FDR ≤ 0.05).

## 4. Discussion

This study demonstrates that dietary patterns are associated with distinct inflammatory- and immune-related plasma proteins in patients with IBD. We showed that plasma levels of FGF-19, a gut-derived hormone regulating bile acid synthesis, were significantly lower in patients adhering to a diet rich in refined carbohydrates and sugar. Conversely, a Mediterranean-style pattern was associated with significantly higher FGF-19 levels. These associations were robust in analyses stratified for IBD type and disease activity, while adjusting for relevant confounders (e.g., ileocecal resection and colectomy). Moreover, a pattern characterised by particularly high alcohol and coffee intake was associated with increased plasma levels of CCL11, but decreased IL12B levels. Taken together, our data demonstrate that dietary intake may modulate specific plasma inflammatory proteins, which has yet remained unexplored in the context of established IBD.

The first identified dietary pattern was characterised by foods high in refined carbohydrates and sugar including bread, spreads, pastry, sugar and sweets, savoury snacks and juice, a diet that is more frequently observed in patients with IBD compared to healthy controls [23]. Several epidemiological studies identified sugar as a risk factor for IBD [25]. In the present study, a higher adherence to a pattern high in sugar and refined carbohydrates was associated with lower levels of plasma FGF-19, a result that was robust throughout all stratified analyses for IBD type, disease activity and protein intake and remained significant after adjusting for surgical history, a known risk factor for impaired FGF-19 signalling [22]. FGF-19 is a protein produced mainly in the ileum upon activation of the Farnesoid X Receptor (FXR), which inhibits hepatic bile acid synthesis [26]. Lower FGF-19 levels have been observed in IBD, especially in CD patients with ileal disease involvement or surgical history [22]. Previous studies have demonstrated that intestinal inflammation, together with impaired intestinal barrier integrity and malabsorption of bile acids, is associated with the decreased release of FGF-19 into the circulation [27,28,29]. Interestingly, decreased fasting FGF-19 levels, reflecting impaired intestinal or hepatic FXR signalling, have also been associated with obesity and the development of metabolic-associated fatty liver disease (MAFLD) [30,31]. The FXR-FGF-19 axis has long been recognised as a therapeutic target, e.g., through pharmacological activation with FXR agonists, also in patients with IBD, particularly those with active inflammation and the concomitant disruption of bile acid metabolism (e.g., after ileocecal resection in patients with CD) [32,33]. Similarly, experimental work indicated that FXR activation could attenuate chemically induced intestinal inflammation, restore intestinal barrier integrity, inhibit the production of pro-inflammatory cytokines and reduce the loss of goblet cells in mice, fuelling the rationale for exploring FXR agonists as therapeutics for IBD [34]. In mice fed a high-sugar Western diet, supplementation with an FXR agonist reduced diet-induced cytokine expression [35]. Moreover, feeding studies in mice have suggested that in contrast to fat, a high carbohydrate meal also modulates the FGF-19 response via additional FXR-independent mechanisms [36].

In combination with our findings, this suggests that lower levels of FGF-19 may constitute a biomarker that is amenable to nutritional intervention. Specifically, patients with IBD and accompanying bile acid derangements may benefit from a higher consumption of fruit, fish, nuts, tea and cereals, foods that constitute a healthy Mediterranean diet and are known for their anti-inflammatory properties related to a high content of omega-3 fatty acids, polyphenols and fibre, while limiting the consumption of meat, refined carbohydrates and sugar.

The fourth dietary pattern was characterised by a high intake of coffee and alcohol, in contrast to a low intake of tea and moderate consumption of spreads and dairy [6]. This pattern was strongly associated with higher levels of plasma CCL11, but with lower levels of plasma IL12B. CCL11, also known as eotaxin-1, is a selective chemoattractant for eosinophils, mediating the activation and recruitment of these cells to the intestinal lamina propria [37,38]. Circulating CCL11 levels have been shown to be elevated in patients with IBD and are associated with inflammatory disease activity [3,39]. In line with our findings, previous studies have found that chronic alcohol consumption is associated with increased plasma CCL11 levels and is reduced upon alcohol discontinuation [40]. Interestingly, a number of antioxidant substances, including flavonoids, have been shown to suppress eotaxin-1 expression via NFκB- and STAT6-mediated signalling in various human cell lines [41]. Moreover, eotaxin-1 is intimately associated with obesity, where increased eotaxin-1 levels have been demonstrated in visceral adipose tissue (VAT), while diet-induced weight loss led to reduction in plasma eotaxin-1 [42]. Patients with IBD, particularly CD, with visceral adiposity are at increased risk of disease complications [43]. A limited intake of alcohol and meat (PC4-5) has a potential to reduce eotaxin-1. In line with these observations, orally administered anti-eotaxin-1 monoclonal antibodies have shown to be able to alleviate immune-mediated hepatitis in mice, suggesting that eotaxin-1 may also be of interest to target in non-alcoholic steatohepatitis (NASH) [44]. Currently, bertilimumab, a humanised monoclonal antibody targeting human eotaxin-1, is under evaluation for UC in a clinical trial (e.g., NCT01671956).

Apart from CCL11, this dietary pattern dominated by a high intake of coffee and alcohol, was associated with lower plasma IL12B levels. Alcohol consumption has previously been demonstrated to inhibit immune system activation by decreasing T-cell activity and IL-12 levels in healthy individuals [45]. In addition, high coffee consumption may also lead to decreased levels of IL-12, as has been reported previously [46]. Interestingly, coffee contains chlorogenic acid (CGA), a compound to which this IL-12-lowering effect has previously been attributed to, since this is consistent with data from experimental studies showing the inhibition of IL-12 secretion upon stimulation of adenosine 2A receptors (of which CGA is a common ligand) [47]. Of note, cigarette smoke exposure is often associated with decreased plasma IL-12B levels, also in patients with IBD [22]. In IBD, the *IL12B* gene, encoding for the p40 subunit of IL-12, is a known IBD susceptibility locus [48]. Lower circulating levels of IL-12B may be reflective of disrupted immunity and impaired antitumor activity, as has earlier been demonstrated for smoking patients with coronary artery disease [49]. Although the exact role of IL-12B in the pathophysiology of IBD remains elusive, it may potentially be relevant given that the IL-12/23 axis forms an established therapeutic target. One could hypothesise that the aggregate of genetic susceptibility, dietary intake and smoking modulates response to anti-IL-12/23 therapies (e.g., the biologic ustekinumab) in patients with IBD.

In this study, we have associated plasma levels of 73 distinct plasma inflammatory proteins to diets identified by an unsupervised pattern analysis in 454 patients with IBD, while correcting for relevant clinical confounders. Several limitations of this study warrant recognition. First, we analysed both protein levels and dietary intake on a cross-sectional level, precluding the assessment of potential causality between dietary intake and protein levels. Future longitudinal studies using dietary recall methods will be relevant to show relationships between dietary intake at the moment of a disease flare. Moreover, controlled dietary intervention studies will be able to link acute and personalized increases in inflammatory markers in response to meals. Efforts are underway to continuously measure inflammatory biomarkers in blood with wearable devices similar to continuous glucose monitors [50,51], and will further elucidate the diet–inflammation relationship. In addition, future functional studies could provide mechanistic validation of the findings and also elucidate the contribution of specific nutrients or bioactive compounds to certain protein level alterations. Second, while we did not detect major differences in our results when stratifying for disease activity, a potential limitation is that disease activity was only assessed by clinical and serological measures and was not endoscopically determined, and that faecal calprotectin levels were insufficiently available at the time of sampling. Third, performing PCA on dietary data necessitates some arbitrary decisions to be made, e.g., with regard to the interpretation of patterns and the number of patterns to retain. Moreover, PCA assesses overall dietary habits in a given population. While stronger correlations with proteins may be observed when analysing specific nutrients such as fatty acids and carbohydrates separately, dietary patterns are of high clinical relevance as they consider nutrient interactions and reflect the patient’s overall dietary profile, i.e., a complex combination of foods rather than single nutrients, which is well captured by PCA [24]. Performing a comprehensive analysis of dietary patterns also allows one to more easily identify dietary risk factors for each individual patient. This makes the assessment of dietary patterns an attractive approach, not only to study the composite influence of diet on systemic inflammation but also to identify patients who might benefit the most from nutritional interventions.

## 5. Conclusions

In this study, four dietary patterns were identified that were significantly associated with the plasma inflammatory protein profile in a real-life cohort of patients with IBD. Our results support the relevance of FXR-FGF-19 signalling in IBD, fuel the rationale for its potential dietary modulation, and suggest a benefit of a healthy Mediterranean-style diet in patients with IBD with bile acid derangements. In addition, we observed a strong association between CCL11/eotaxin-1 levels and a dietary pattern dominated by high intake of coffee and alcohol, which may reflect a relationship between these food groups and intestinal inflammation. Although the presented findings warrant further validation, our study convincingly demonstrated that it is important to take dietary intake into account when querying proteomic data for biomarker discovery and supports both pro-inflammatory and anti-inflammatory roles of diet in the context of IBD.

## Figures and Tables

**Table 1:** Demographic and clinical characteristics of the full study population and for patients with CD and UC separately.

Variable	Total	CD	UC	*p*-Value
	*n* = 454	*n* = 264	*n* = 190	
Age (years)	41.4 ± 14.4	39.2 ± 14.1	44.5 ± 14.2	<0.01
Sex, *n* (%)				<0.01
*Male*	173 (38.1)	87 (33.0)	86 (45.3)	
*Female*	281 (61.9)	177 (67.0)	104 (54.7)	
BMI (kg/m^2^)	24.7 [21.9–28.1]	23.9 [21.4–27.6]	25.5 [22.8–29.1]	<0.01
Current smoking, *n* (%)	441 (97.1)	257 (97.3)	184 (96.8)	<0.01
*Yes*	93 (20.5)	73 (28.4)	20 (10.9)	
*No*	348 (76.7)	184 (71.6)	164 (89.1)	
**Montreal classification**				
Montreal Age (A), *n* (%)	453 (99.8)	263 (99.6)	190 (100)	<0.01
*A1 (≤16 years)*	58 (12.8)	42 (16.0)	16 (8.4)	
*A2 (17–40 years)*	298 (65.6)	179 (68.1)	119 (62.6)	
*A3 (>40 years)*	97 (21.4)	42 (16.0)	55 (28.9)	
Montreal Location (L), *n* (%)	-	264 (100)	-	
*L1 (ileal disease)*	-	92 (34.8)	-	
*L2 (colonic disease)*	-	58 (22.0)	-	
*L3 (ileocolonic disease)*	-	91 (34.5)	-	
*L4 (upper GI disease)*	-	5 (1.9)	-	
*L1 + L4*	-	8 (3.0)	-	
*L2 + L4*	-	6 (2.3)	-	
*L3 + L4*	-	4 (1.5)	-	
Montreal Behaviour (B), *n* (%)	-	264 (100)	-	
*B1 (non-stricturing, non-penetrating)*	-	105 (39.8)	-	
*B2 (stricturing)*	-	50 (18.9)	-	
*B3 (penetrating)*	-	24 (9.1)	-	
*B1 + P (perianal disease)*	-	32 (12.1)	-	
*B2 + P (perianal disease)*	-	39 (14.8)	-	
*B3 + P (perianal disease)*	-	14 (5.3)	-	
Montreal Extension (E), *n* (%)	-	-	187 (98.4)	
*E1 (proctitis)*	-	-	29 (15.5)	
*E2 (left-sided colitis)*	-	-	61 (32.6)	
*E3 (pancolitis)*	-	-	97 (51.9)	
**Medication use**				
Aminosalicylates, *n* (%)	158 (34.8)	27 (10.2)	131 (68.9)	<0.01
Thiopurines, *n* (%)	169 (37.2)	111 (42.0)	58 (30.5)	0.01
Steroids, *n* (%)	106 (23.3)	65 (24.6)	41 (21.6)	0.45
Calcineurin inhibitors, *n* (%)	8 (1.8)	2 (0.8)	6 (3.2)	0.06
Methotrexate, *n* (%)	32 (7.0)	28 (10.6)	4 (2.1)	<0.01
TNF-α-antagonists, *n* (%) ^†^	115 (25.3)	100 (37.9)	15 (7.9)	<0.01
**Disease activity**				
HBI (CD), *n* (%)	-	252 (95.5)	-	
<5	-	161 (63.9)	-	
≥5	-	91 (36.1)	-	
SCCAI (UC), *n* (%)	-	-	183 (96.3)	
≤2	-	-	130 (71.4)	
>2	-	-	53 (28.6)	
CRP, *n* (%)	382 (84.1)	224 (84.8)	158 (83.2)	<0.05
≤5 mg/L	279 (61.5)	153 (68.3)	126 (79.7)	
>5 mg/L	103 (22.7)	71 (31.7)	32 (20.3)	
**Surgical history**				
Ileocecal resection, *n* (%)	87 (19.2)	87 (33.0)	0 (0.0)	<0.01
Colon resection (or partial), *n* (%)	76 (16.7)	43 (16.3)	33 (17.4)	0.76

Data are presented as proportions *n* with corresponding percentages (%), means ± standard deviation (SD) or as medians (1st–3rd quartile, or interquartile range, IQR) in case of continuous variables. *p*-values ≤ 0.05 were considered statistically significant. ^†^ The use of TNF-α-antagonists included use of the following compounds: infliximab, adalimumab, golimumab and certolizumab pegol. Abbreviations: BMI, body-mass index; CD, Crohn’s disease; CRP, C-reactive protein; HBI, Harvey–Bradshaw index; IBD, inflammatory bowel disease; SCCAI, simple clinical colitis activity index; TNF-α, tumour necrosis factor alpha; UC, ulcerative colitis. ‘-’: Not applicable.

**Table 2:** Habitual dietary intake of the full cohort and for patients with CD and UC separately.

	IBD	CD	UC	*p*-Value
	*n* = 454	*n* = 264	*n* = 190	
**Macronutrients intake per day**				
Energy intake (kcal/day)	1,824 (1,519;2,258)	1,780 (1,480;2,194)	1,917 (1,552;2,364)	0.059
EI/BMR	1.14 (0.93–1.42)	1.17 (0.93–1.42)	1.11 (0.93–1.41)	0.994
Total protein (g/day)	65.7 (54.3;80.5)	63.6 (52.2–76.5)	70.6 (57.5–83.5)	0.001 *
*Energy (%)*	14.3 (12.7–15.9)	14.2 (12.7–15.6)	14.5 (12.7–16.2)	0.050
Protein (g/kg)	0.87 (0.70–1.06)	0.89 (0.70–1.07)	0.84 (0.70–1.06)	0.768
Plant protein (g/day)	27.3 (22.2;34.6)	26.2 (21.4–33.3)	28.1 (23.2–35.8)	**0.015**
Animal protein (g/day)	37.9 (29.4;47.6)	36.1 (28.2–45.8)	41.1 (32.2–49.5)	0.003 *
Total fat (g/day)	72.1 (57.2;90.7)	69.3 (55.4–89.5)	75.2 (59.7–98.2)	**0.038**
*Fat Energy (%)*	35.7 (31.8–39.4)	35.6 (31.6–39.1)	36.0 (32.3–39.9)	0.275
Carbohydrates (g/day)	208 (165;267)	211 (161–261)	207 (171–283)	0.405
*Carbohydrate Energy (%)* ^†^	45.9 (41.8–49.7)	46.3 (42.5–50.4)	45.6 (41.3–48.6)	**0.028**
Alcohol (g/day)	1.3 (0.0;5.9)	1.2 (0.0–5.0)	1.5 (0.0–6.7)	0.323
*Alcohol Energy (%)*	0.5 (0.0–2.0)	0.4 (0.0–1.9)	0.6 (0.0–2.0)	0.467
**Food groups (g/day)**				
Alcoholic beverages	13.4 (0.0–71.1)	11.9 (0.0–63.9)	16.2 (0.0–77.6)	0.276
Breads	130 (80.0–164)	127 (77.4–159)	133 (90.1–174)	0.069
Cereals	0.0 (0.0–3.5)	0.0 (0.0–2.9)	0.0 (0.0–4.8)	0.309
Cheese	21.1 (8.5–36.9)	20.7 (8.1–33.0)	21.8 (9.2–38.9)	0.236
Coffee	232 (17.9–465)	232 (11.1–465)	232 (22.3–465)	0.925
Dairy	182 (96.5–330)	159 (81.9–298)	242 (123–348)	0.001 *
Eggs	8.9 (4.5–17.9)	8.9 (4.5–17.9)	8.9 (4.5–17.9)	0.931
Fish	11.1 (4.4–17.7)	11.0 (4.2–17.3)	11.3 (4.9–18.3)	0.271
Fruits	110(42.3–220)	84.6 (42.3–220)	119 (50.9–220)	**0.020**
Juice	26.7 (0.0–107)	26.7 (0.0–139)	21.5 (0.0–96.5)	0.056
Legumes	2.2 (0.0–11.0)	0.0 (0.0–11.0)	4.4 (0.0–16.4)	**0.021**
Meat	86.1 (58.5–111)	84.1 (49.7–109)	91.6 (65.9–113)	0.082
Non-alcoholic beverages	104 (20.9–278)	136 (26.2–284)	52.9 (13.0–271)	<0.001 *
Nuts	5.4 (1.8–13.2)	4.3 (1.4–12.6)	6.7 (2.1–14.1)	0.063
Pasta	12.7 (7.9–25.5)	12.7 (7.9–25.5)	15.9 (7.9–31.8)	0.065
Pastry	23.8 (12.4–40.3)	21.8 (11.3–39.0)	26.7 (14.7–44.2)	0.015
Potatoes	72.3 (40.8–111)	71.3 (39.6–104)	85.6 (41.3–119)	0.181
Prepared meals	32.0 (12.9–58.8)	32.4 (12.9–59.9)	27.6 (12.9–54.2)	0.265
Rice	14.9 (4.7–24.8)	14.9 (4.0–24.8)	14.9 (5.5–24.8)	0.574
Sauces	10.0 (4.6–20.8)	10.5 (4.3–21.3)	9.2 (4.7–20.5)	0.860
Savoury snacks	13.1 (5.4–23.9)	13.4 (5.1–24.7)	12.8 (5.8–23.6)	0.760
Soup	35.8 (9.0–71.5)	35.8 (9.0–44.5)	35.8 (15.8–71.5)	0.335
Spreads	20.6 (8.3–31.4)	19.1 (7.1–30.8)	22.5 (9.5–32.6)	0.146
Sugar/Sweets	29.8 (14.1–49.6)	30.3 (12.9–48.7)	28.9 (15.2–50.0)	0.916
Tea	232 (44.6–465)	232 (44.6–348)	232 (44.6–465)	0.841
Vegetables	107 (62.4–147)	81.8 (61.5–114)	108 (62.8–151)	0.225

Data are presented as medians with interquartile ranges (1st–3rd quartile). *p*-values ≤ 0.05 were considered statistically significant. ^†^ Intake reported as percentage of total energy intake, calculated as (macronutrient/total energy intake) ∗ 100%. **Bold** *p*-values indicate nominally statistically significant differences between CD and UC, and asterisks (*) indicate statistical significance under a false discovery rate (FDR) of 5%.

**Table 3:** Characteristics of dietary patterns derived from PCA with *varimax* rotation.

	PC1	PC2	PC3	PC4	PC5
Alcohol	−0.150	−0.001	**0.366**	**0.494**	−0.037
Breads	**0.703**	0.065	0.025	0.249	0.146
Cereals	0.050	**0.343**	0.049	0.121	−0.170
Cheese	0.047	0.156	0.190	0.264	0.209
Coffee	0.039	0.066	−0.079	**0.672**	0.129
Dairy	0.288	0.133	−0.246	**0.304**	0.066
Eggs	0.184	**0.382**	0.237	−0.155	0.180
Fish	−0.038	**0.560**	−0.012	−0.029	0.114
Fruit	−0.015	**0.632**	−0.264	−0.087	0.105
Juice	**0.338**	−0.109	−0.065	−0.116	0.028
Legumes	0.009	0.061	0.146	0.016	**0.514**
Meat	0.257	**−0.386**	−0.001	0.189	**0.550**
Non-alcoholic beverages	0.257	**−0.466**	0.138	−0.257	0.065
Nuts	0.031	**0.426**	**0.351**	−0.026	−0.072
Pasta	0.032	0.028	**0.655**	0.074	0.219
Pastry	**0.585**	0.102	0.170	−0.168	0.060
Potatoes	0.290	−0.247	−0.079	0.136	**0.651**
Prepared meals	0.163	−0.062	**0.519**	0.143	−0.263
Rice	−0.104	0.201	**0.402**	−0.157	0.204
Sauces	0.249	−0.164	**0.659**	0.036	0.130
Savoury snacks	**0.510**	−0.228	**0.325**	−0.195	0.101
Soup	0.110	0.242	0.097	0.180	0.256
Spreads	**0.707**	0.087	−0.009	**0.325**	0.090
Sugar/Sweets	**0.532**	−0.007	0.106	0.024	−0.145
Tea	0.002	**0.366**	−0.051	**−0.572**	0.107
Vegetables	−0.173	0.258	0.058	−0.119	**0.659**

Table shows the contribution of food groups per dietary pattern (principal component). Values indicate the positive and negative factor loadings of food groups based on their correlation with each principal component. Values in **bold** indicate factor loadings above 0.3 (for high intakes) and below −0.3 (for low intakes).

## Data Availability

The dataset(s) used for the current study are available from the corresponding author upon reasonable request. The data for the Groningen 1000IBD cohort can be requested at the European Genome-Phenome Archive data repository with the accession number: EGAS00001002702.

## Supplementary Materials

The following supporting information can be downloaded at: https://www.mdpi.com/article/10.3390/nu14122522/s1, Figure S1: Scree plot for the determination of PCs to retain from PCA. Eventually, five PCs were selected based on their eigenvalues (>1) and interpretability; Figure S2: Correlations of food groups with principal components for the determination of eigenvectors. PC, principal component derived from PCA with varimax rotation. Colour red indicates a positive correlation, blue indicates a negative correlation. Colour density visualizes the strength of the correlation, which is an indicator of how highly a food group is loaded in the PC (See Table 3). Table S1: Abbreviations, full names, UniProt IDs and detection rates of all 92 plasma proteins measured using the Olink^®^ Inflammation panel; Table S2: Composition of 26 food groups included in the PCA; Table S3: Characteristics of participants with implausible FFQs; Table S4: Robust PCA: Factor loadings of food groups based on its underlying correlation matrix demonstrating the individual contribution of food groups to each of the identified dietary patterns; Table S5: Results of the permutation analysis of variance; Table S6: PC loadings (mean (SD)) in patients with and without surgical history (including ileocecal resection and colectomy) and in patients with and without active disease at time of sampling. Table S7: Results of the multivariate association analyses performed between dietary patterns and plasma protein levels.
