# Supplementary material for: Long-Term Dietary Patterns Are Reflected in the Plasma Inflammatory Proteome of Patients with Inflammatory Bowel Disease
Source: Nutrients. 2022 Jun 17;14(12):2522. doi: 10.3390/nu14122522 (PMC9228369; doi:10.3390/nu14122522)
Supplement: Supplementary file 1 [file nutrients-14-02522-s001.zip › nutrients-1756673-supplementary/nutrients-1756673-supplementary-xml.pdf]

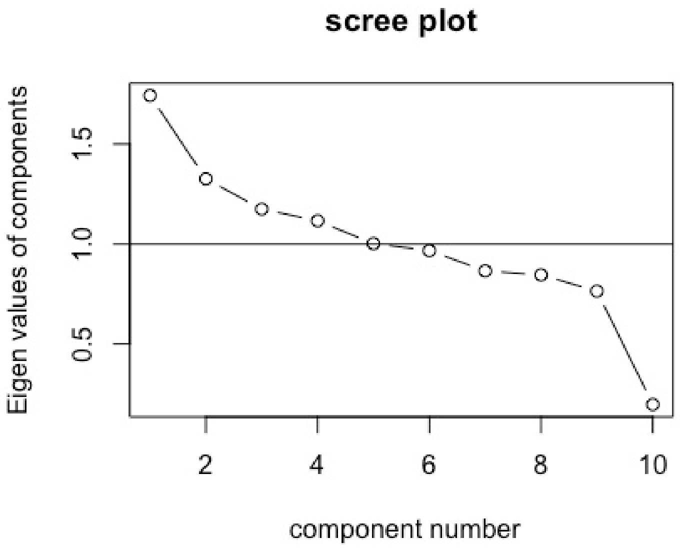

Figure S1: Scree plot for the determination of PCs to retain from PCA. Eventually, five PCs were selected based on their eigenvalues (>1) and interpretability.

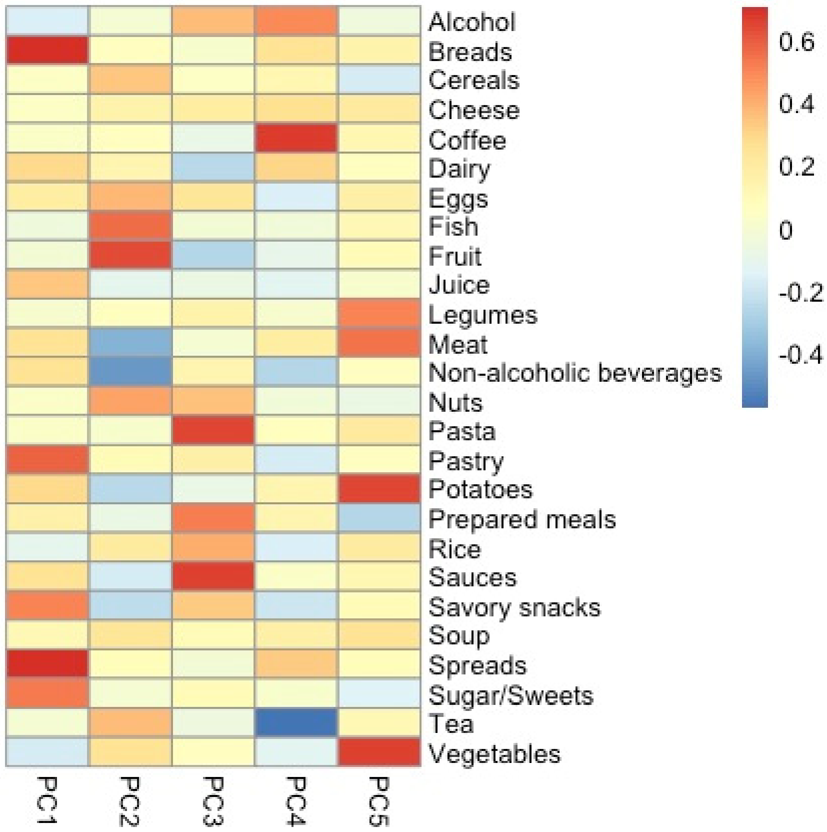

Figure S2: Correlations of food groups with principal components for the determination of eigenvectors. PC, principal component derived from PCA with varimax rotation. Colour red indicates a positive correlation, blue indicates a negative correlation. Colour density visualizes the strength of the correlation, which is an indicator of how highly a food group is loaded in the PC (See Table 3).

Supplementary Table S1. Abbreviations, full names, UniProt IDs and detection rates of all 92 plasma proteins measured using the Olink® Inflammation panel.

Abbreviation	Full name	UniProt ID	% IBD	% CD	% UC	Inclusion
			n=454	*n*=264	*n*=190	
4E-BP1	Eukaryotic translation initiation factor 4E-binding protein 1	Q13541	100%	100%	100%	Yes
ADA	Adenosine Deaminase	P00813	100%	100%	100%	Yes
ARTN	Artemin	Q5T4W7	27.9%	29.5%	23.2%	No
AXIN1	Axin-1	O15169	100%	100%	100%	Yes
β-NGF	Beta-nerve growth factor	P01138	100%	100%	100%	Yes
CASP-8	Caspase-8	Q14790	100%	100%	100%	Yes
CCL11	Eotaxin	P51671	100%	100%	100%	Yes
CCL19	C-C motif chemokine 19	Q99731	100%	100%	100%	Yes
CCL20	C-C motif chemokine 20	P78556	100%	100%	100%	Yes
CCL23	C-C motif chemokine 23	P55773	100%	100%	100%	Yes
CCL25	C-C motif chemokine 25	O15444	100%	100%	100%	Yes
CCL28	C-C motif chemokine 28	Q9NRJ3	100%	100%	100%	Yes
CCL3	C-C motif chemokine 3	P10147	100%	100%	100%	Yes
CCL4	C-C motif chemokine 4	P13236	100%	100%	100%	Yes
CD244	Natural killer cell receptor 2B4	Q9BZW8	100%	100%	100%	Yes
CD40	CD40L receptor	P25942	100%	100%	100%	Yes
CD5	T-cell surface glycoprotein CD5	P06127	100%	100%	100%	Yes
CD6	T-cell surface glycoprotein CD6 isoform	P30203	100%	100%	100%	Yes
CD8A	T-cell surface glycoprotein CD8 alpha chain	P01732	100%	100%	100%	Yes
CDCP1	CUB domain-containing protein 1	Q9H5V8	100%	100%	100%	Yes
CSF-1	Macrophage colony-stimulating factor 1	P09603	100%	100%	100%	Yes
CST5	Cystatin D	P28325	100%	100%	100%	Yes
CX3CL1	Fractalkine	P78423	100%	100%	100%	Yes
CXCL1	C-X-C motif chemokine 1	P09341	100%	100%	100%	Yes
CXCL10	C-X-C motif chemokine 10	P02778	100%	100%	100%	Yes
CXCL11	C-X-C motif chemokine 11	O14625	100%	100%	100%	Yes
CXCL5	C-X-C motif chemokine 5	P42830	100%	100%	100%	Yes
CXCL6	C-X-C motif chemokine 6	P80162	100%	100%	100%	Yes
CXCL9	C-X-C motif chemokine 9	Q07325	100%	100%	100%	Yes
DNER	Delta and Notch-like epidermal growth factor-related receptor	Q8NFT8	100%	100%	100%	Yes
EN-RAGE	Protein S100-A12	P80511	99.6%	99.2%	100%	Yes
FGF-19	Fibroblast growth factor 19	O95750	100%	100%	100%	Yes
FGF-21	Fibroblast growth factor 21	Q9NSA1	99.6%	100%	98.9%	Yes
FGF-23	Fibroblast growth factor 23	Q9GZV9	97.6%	98.5%	96.3%	Yes
Flt3L	Fms-related tyrosine kinase 3 ligand	P49771	100%	100%	100%	Yes
GDNF	Glial cell line-derived neurotrophic factor	P39905	97.8%	96.6%	99.5%	Yes
HGF	Hepatocyte growth factor	P14210	100%	100%	100%	Yes
IFN-γ	Interferon gamma	P01579	99.6%	100%	98.9%	Yes

IL-10	Interleukin-10	P22301	100%	100%	100%	Yes
IL-10RA	Interleukin-10 receptor subunit alpha	Q13651	68.5%	70.5%	65.8%	Yes
IL-10RB	Interleukin-10 receptor subunit beta	Q08334	100%	100%	100%	Yes
IL-12B	Interleukin-12 subunit beta	P29460	100%	100%	100%	Yes
IL-13	Interleukin-13	P35225	23.6%	24.2%	22.6%	No
IL-15RA	Interleukin-15 receptor subunit alpha	Q13261	95.6%	95.8%	95.3%	Yes
IL-17A	Interleukin-17A	Q16552	94.9%	96.2%	93.2%	Yes
IL-17C	Interleukin-17C	Q9P0M4	61.7%	62.9%	60.0%	Yes
IL-18	Interleukin-18	Q14116	100%	100%	100%	Yes
IL-18R1	Interleukin-18 receptor 1	Q13478	100%	100%	100%	Yes
IL-20RA	Interleukin-20 receptor subunit alpha	Q9UHF4	49.8%	51.5%	47.4%	Yes
IL-24	Interleukin-24	Q13007	17.8%	16.3%	20.0%	No
IL-2RB	Interleukin-2 receptor subunit beta	P14784	24.7%	23.1%	26.8%	No
IL-4	Interleukin-4	P05112	18.9%	16.7%	22.1%	No
IL-5	Interleukin-5	P05113	48.0%	50.8%	44.2%	Yes
IL-6	Interleukin-6	P05231	100%	100%	100%	Yes
IL-7	Interleukin-7	P13232	100%	100%	100%	Yes
IL-8	Interleukin-8	P10145	100%	100%	100%	Yes
LAP TGF-β-1	Latency-associated peptide transforming growth factor beta-1	P01137	100%	100%	100%	Yes
LIF-R	Leukaemia inhibitory factor receptor	P42702	99.8%	99.6%	100%	Yes
MCP-1	Monocyte chemotactic protein 1	P13500	100%	100%	100%	Yes
MCP-2	Monocyte chemotactic protein 2	P80075	100%	100%	100%	Yes
MCP-3	Monocyte chemotactic protein 3	P80098	74.2%	75.4%	72.6%	Yes
MCP-4	Monocyte chemotactic protein 4	Q99616	100%	100%	100%	Yes
MMP-1	Matrix metalloproteinase-1	P03956	100%	100%	100%	Yes
MMP-10	Matrix metalloproteinase-10	P09238	100%	100%	100%	Yes
NRTN	Neurturin	Q99748	12.3%	12.5%	12.1%	No
NT-3	Neurotrophin-3	P20783	100%	100%	100%	Yes
OPG	Osteoprotegerin	O00300	100%	100%	100%	Yes
OSM	Oncostatin-M	P13725	100%	100%	100%	Yes
PD-L1	Programmed cell death 1 ligand 1	Q9NZQ7	100%	100%	100%	Yes
SCF	Stem cell factor	P21583	100%	100%	100%	Yes
SIRT2	SIR2-like protein 2	Q8IXJ6	100%	100%	100%	Yes
SLAMF1	Signalling lymphocytic activation molecule	Q13291	100%	100%	100%	Yes
ST1A1	Sulfotransferase 1A1	P50225	100%	100%	100%	Yes
STAMBP	STAM-binding protein	O95630	99.8%	99.6%	100%	Yes
TGF-α	Transforming growth factor alpha	P01135	100%	100%	100%	Yes
TNFB	TNF-beta	P01374	100%	100%	100%	Yes
TNFRSF9	Tumour necrosis factor receptor superfamily member 9	Q07011	100%	100%	100%	Yes
TNFSF14	Tumour necrosis factor ligand superfamily member 14	O43557	100%	100%	100%	Yes

TRAIL	TNF-related apoptosis-inducing ligand	P50591	100%	100%	100%	Yes
TRANCE	TNF-related activation-induced cytokine	O14788	100%	100%	100%	Yes
TWEAK	Tumour necrosis factor (Ligand) superfamily, member 12	O43508	100%	100%	100%	Yes
uPA	Urokinase-type plasminogen activator	P00749	100%	100%	100%	Yes
VEGF-A	Vascular endothelial growth factor A	P15692	100%	100%	100%	Yes

Supplementary Table S2. Composition of 26 food groups included in the PCA.

No.	Food group	Food items
#1	Alcohol	Red wine<br>White wine<br>Fortified wines<br>Beer<br>Spirit drinks<br>Other types of alcoholic drinks
#2	Breads	Pain rusks/crispbread/crackers<br>Croissants/other breads<br>Slices of bread/sandwich bread
#3	Cereals	Granola/muesli/cereals
#4	Cheese	Cheese(/spread) $20^{+}/30^{+}$<br>Cheese (/spread) 40^{+}<br>Cheese (/spread) 48^{+}<br>Cheese added to dinner<br>Cheese in-between meals<br>Cream cheese/foreign cheeses
#5	Coffee	Coffee
#6	Dairy	Reduced-fat/semi-skimmed milk<br>Whole milk<br>Buttermilk<br>Low-fat/skimmed milk<br>Quark/curd/fruitcurd<br>Drinking yoghourt/milk drink with flavour and sugar<br>Low-fat (fruit) yoghourt<br>Low-fat unsweetened yoghourt<br>Whole natural yoghourt<br>Other types of puddings, custard, yoghourt and quark/curd<br>Chocolate milk<br>Other types of dairy drinks<br>Ready-made breakfast drinks<br>Ice cream/milk-based ice<br>Ready-made pap/porridge<br>Whole pudding

		Whipped cream<br>Reduced-fat coffee milk<br>Coffee creamer<br>Whole fat coffee milk<br>Ordinary milk added to coffee<br>Other types of coffee milk
#7	Eggs	Boiled eggs<br>Fried eggs
#8	Fish	Low-fat fish/white fish<br>Fatty fish<br>Salty herring<br>Other types of fish<br>Fried haddock filet/fried cod bits<br>Fish home-cooked with fat (fish prepared fat)
#9	Fruits	Whole fruits
#10	Juice	Fruit juices
#11	Legumes	Legumes
#12	Meat	Sausage/bacon (cold cuts)<br>Other types of cold cut meat and sausages<br>Mince meat<br>Smoked sausage<br>Steak/roast beef/ox meat<br>Meat olives/braised meat<br>Pork steak/fried rice meat/schnitzel<br>Pork fillet/pork chops<br>Pork belly/bacon<br>Chicken<br>Other types of meats or poultry<br>Gravy<br>Cold meats and sausages (cold cuts) in-between meals
#13	Non-alcoholic beverages	Non-alcoholic beer<br>Soda/lemonade with sugar<br>Light sodas/lemonade without sugar
#14	Nuts	Peanut butter<br>Peanuts and nuts added to dinner<br>Peanuts and nuts in-between meals
#15	Pasta	Pasta
#16	Pastry	Small cookies/biscuits<br>Cake/large cookies<br>Pastry/pie<br>Gingerbread/cake bars/food biscuits
#17	Potatoes	Cooked potatoes/mashed potatoes<br>French fries/baked potatoes
#18	Prepared meals	Other types of ready-made meals<br>Chinese or Indian ready-made meals<br>Fast food<br>Pizza
#19	Rice	Rice
#20	Sauces	Hot sauces

		Salad dressing with and without oil<br>Mayonnaise<br>Low-fat mayonnaise/ mayonnaise/non-red sauces/condiments<br>Mayonnaise added to snacks<br>Low-fat mayonnaise /mayonnaise/non-red sauces/condiments added to snacks
#21	Savoury snacks	Sandwich spreads/salads (on bread)<br>Warm savoury snacks<br>Potato crisps/salty snacks/pretzels<br>Sandwich spreads/salads on French bread/toast
#22	Soup	Soups with legumes<br>Soups without legumes
#23	Spreads	Butter or margarine (for bread)<br>Low-fat margarine (for bread)<br>Other types of margarine (for bread)
#24	Sugar/Sweets	Chocolate spreads/sprinkles (for bread)<br>Other types of sweet spread/sprinkles (for bread)<br>Sugar/syrup (for yoghourt)<br>Sugar (for coffee)<br>Sugar/honey (for tea)<br>Candy bars<br>Chocolate<br>Candy
#25	Tea	Tea
#26	Vegetables	Boiled vegetables with butter<br>Boiled vegetables without butter<br>Stir-fried vegetables

Supplementary Table S3. Characteristics of participants with implausible FFQs

Outlier	Age	Sex	BMI	BMR	Total kcal	EI/BMR ratio	Protein g/day	Protein per kg weight	Excluded from analysis
1	39	Female	27	1543.2	*NA*	*NA*	*NA*	*NA*	Yes
2	51	Female	25	1452.5	*NA*	*NA*	*NA*	*NA*	Yes
3	51	Male	27	2014.7	*NA*	*NA*	*NA*	*NA*	Yes
4	43	Male	28	2101.2	6805	3.2	162.4	1.6	No
5	44	Male	17	1524.5	5546	3.6	136.8	2.3	Yes
6	53	Male	31	1983.7	4036	2.0	130.6	1.3	No
7	64	Male	22	1482.0	4173	2.8	101.7	1.5	Yes

8	30	Male	23	1885.2	5578	3.0	137	1.7	No
9	44	Male	22	1819.5	4103	2.3	136.7	1.7	No
10	62	Male	20	1144.2	6791	5.9	212.7	4.1	Yes
11	30	Female	23	1398.3	3963	2.8	120.9	2.0	Yes
12	19	Female	20	1447.3	4097	2.8	134.1	2.3	Yes
13	56	Female	21	1204.2	432	0.4	16.5	0.3	Yes
14	33	Female	24	1475.6	489	0.3	34.6	0.5	Yes
15	41	Female	19	1291.0	480	0.4	17.9	0.3	Yes
16	39	Female	30	1857.5	319	0.2	2	0.0	Yes
17	46	Female	31	1592.8	466	0.3	18.1	0.2	Yes

BMR, basal metabolic rate; bmi, body mass index; EI/BMR, ratio of energy intake (kilocalories) to basal metabolic rate estimated by Harris-Benedict equations, kg, kilogram body weight. NA, not applicable.

Supplementary Results

Patterns derived from the robust PCA demonstrated a proportional variance of 5.5%, 7.1%, 6.4%, 4.9% and 5.9%, respectively, cumulatively explaining 29.8% of the variation. We observed a pattern high in potatoes, meat and vegetables (RC1) that was similar to PC5 in the original PCA. PC3 from the original PCA divided into two components in the robust PCA: Namely a pattern low in fruit and dairy and higher in sauces, savoury snacks and non-alcoholic drinks (RC2), that resembled PC3 and a pattern with high loadings of sauces, pasta and cheese (RC4), that also resembled PC3. RC3 was very high in coffee, followed by alcohol and dairy but low in tea, which is similar to PC4. RC5 was high in spreads, bread and sugar and sweets, similar to PC1.

Supplementary Table S4. Robust PCA: Factor loadings of food groups based on its underlying correlation matrix demonstrating the individual contribution of food groups to each of the identified dietary patterns.

	**RC1**	**RC2**	**RC3**	**RC4**	**RC5**
Alcohol	-0.099	0.060	**0.131**	0.158	-0.018
Breads	-0.047	-0.022	-0.066	0.015	**0.478**
Cereals	0.025	-0.071	0.020	0.072	-0.109
Cheese	-0.127	-0.049	0.116	**0.447**	0.164
Coffee	0.009	-0.156	**0.445**	0.045	0.054
Dairy	0.228	-0.224	0.134	-0.077	0.046
Eggs	0.024	-0.030	-0.042	0.169	-0.043
Fish	-0.014	-0.115	-0.009	0.262	-0.111
Fruit	0.032	**-0.373**	-0.137	0.092	0.032

Juice	0.106	-0.035	0.020	-0.034	-0.007
Legumes	0.156	-0.056	0.021	0.037	-0.082
Meat	**0.417**	0.078	0.030	-0.069	-0.005
Non-alcoholic beverages	0.252	0.225	-0.054	-0.052	-0.091
Nuts	-0.101	0.018	-0.002	0.112	0.045
Pasta	-0.014	0.170	-0.104	**0.344**	0.052
Pastry	0.119	-0.009	-0.120	-0.018	0.134
Potatoes	**0.471**	-0.031	0.024	-0.048	-0.027
Prepared meals	-0.103	0.188	0.026	0.097	0.015
Rice	0.022	0.070	-0.141	0.180	-0.041
Sauces	0.033	0.289	-0.027	**0.303**	-0.007
Savory snacks	0.092	0.243	-0.057	0.107	-0.001
Soup	0.136	-0.094	0.016	0.207	-0.050
Spreads	-0.091	-0.028	0.019	0.003	**0.491**
Sugar/Sweets	0.018	0.024	-0.082	-0.290	**0.317**
Tea	0.033	-0.146	**-0.515**	0.005	0.109
Vegetables	**0.332**	-0.094	-0.127	0.218	-0.175

Bold coefficients are < -0.3 or > 0.3 and were considered relevant contributions (see **Methods**).

Supplementary Table S5. Results of the permutation analysis of variance

PC	SS	MS	FModel	R^2	*P*-value	FDR
PC4	103.0716	103.0716	2.3690	0.0052	0.0188	0.0940
PC5	82.2424	82.2424	1.8883	0.0042	0.0516	0.0948
PC1	80.5609	80.5609	1.8495	0.0041	0.0569	0.0948
PC2	67.7659	67.7659	1.5548	0.0034	0.1024	0.1280
PC3	44.6181	44.6181	1.0225	0.0023	0.3532	0.3532

Abbreviations: FDR, false discovery rate; MS, mean of squares; R^2, coefficient of explained variance; SS, sum of squares.

Supplementary Table S6. PC-loadings (mean (SD)) in patients with and without surgical history (including ileocecal resection and colectomy) and in patients with and without active disease.

Dietary pattern	History of ileocecal resection	No previous ileocecal resection	*P*-value	FDR
PC1	0.21 (2.55)	-0.05 (2.64)	0.151	0.252
PC2	-0.11 (2.37)	0.03 (2.00)	0.127	0.318
PC3	-0.11 (1.67)	0.03 (2.16)	0.427	0.458
PC4	-0.20 (1.71)	0.05 (1.75)	0.127	0.272
PC5	-0.12 (1.67)	0.03 (2.06)	0.412	0.475
**Dietary pattern**	**History of colectomy**	**No previous colectomy**	***P*-value**	**FDR**
PC1	0.56 (2.79)	-0.11 (2.58)	0.041	0.154
PC2	-0.41 (2.09)	0.08 (2.06)	0.039	0.195
PC3	-0.04 (2.03)	0.01 (2.09)	0.478	0.478
PC4	0.17 (1.91)	-0.03 (1.71)	0.246	0.335
PC5	0.24 (2.03)	-0.05 (1.98)	0.175	0.263
**Dietary pattern**	**Active disease**	**Remission**	***P*-value**	**FDR**
PC1	-0.27 (2.62)	0.23 (2.63)	0.009	0.068
PC2	0.02 (2.18)	0.01 (2.00)	0.290	0.363

PC3	-0.13 (2.06)	0.16 (2.11)	0.057	0.171
PC4	-0.20 (1.75)	0.18 (1.75)	0.008	0.120
PC5	-0.05 (2.03)	0.08 (1.99)	0.137	0.257

Abbreviations: FDR, false discovery rate.

Supplementary Table S7 (Provided as external data file): Results of the multivariate association analyses performed between dietary patterns and plasma protein levels.
